# DMT-Induced Shifts in Criticality Correlate with Self-Dissolution

**DOI:** 10.1523/JNEUROSCI.0344-25.2025

**Published:** 2025-11-24

**Authors:** Mona Irrmischer, Marco Aqil, Lisa Luan, Tongyu Wang, Hessel Engelbregt, Robin Carhart-Harris, Klaus Linkenkaer-Hansen, Christopher Timmermann

**Affiliations:** ^1^GGZ Research, Academic Center for Trauma and Personality, Amsterdam 1054 GE, The Netherlands; ^2^DMT Research Group, Centre for Psychedelic Research, Imperial College London, London SW7 2AZ, United Kingdom; ^3^Department of Integrative Neurophysiology, Center for Neurogenomics and Cognitive Research (CNCR), Amsterdam Neuroscience, Vrije Universiteit Amsterdam, Amsterdam 1081 HZ, The Netherlands; ^4^Weill Institute for Neurosciences, University of California San Francisco, San Francisco 94158, California; ^5^UCL Centre for Consciousness Research, Experimental Psychology, University College London, London WC1H 0AP, UK

**Keywords:** brain oscillations, criticality, DMT, EEG, self-dissolution

## Abstract

Psychedelics profoundly alter subjective experience and brain dynamics. Brain oscillations express signatures of near-critical dynamics, relevant for healthy function. Alterations in the proximity to criticality have been suggested to underlie the experiential and neurological effects of psychedelics. Here, we investigate the effects of a psychedelic substance (DMT) on the criticality of brain oscillations, and in relation to subjective experience, in humans of either sex. We find that DMT shifts the dynamics of brain oscillations away from criticality in alpha and adjacent frequency bands. In this context, entropy is increased while complexity is reduced. We find that the criticality-shifts observed in alpha and theta bands correlate with the intensity ratings of self-dissolution, a hallmark of psychedelic experience. Finally, using a recently developed metric, the functional excitatory-inhibitory ratio, we find that the DMT-induced criticality-shift in brain oscillations is toward subcritical regimes. These findings have major implications for the neuronal understanding of the self and psychedelics, as well as for the neurological basis of altered states of consciousness.

## Significance Statement

Criticality is characterized by fluctuations occurring on a wide range of spatiotemporal scales and high complexity. Here, we investigate the effects of DMT, a classic psychedelic, on criticality of brain oscillations and in relation to subjective experience. We find that DMT shifts the normally dominant alpha oscillations toward a quieter subcritical state, increasing entropy while reducing complexity, and that this shift correlates with intensity of disruption of the sense of self.

## Introduction

Classic psychedelics elicit a wide range of changes in subjective experience and brain dynamics (Halberstadt, 2015; Nichols, 2016; Vollenweider and Preller, 2020; Kelmendi et al., 2022). In recent years, psychedelics have reemerged at the forefront of scientific and clinical inquiry due to their therapeutic potential and opportunities for research in brain function (Kyzar et al., 2017; Inserra et al., 2021; Kwan et al., 2022; van Elk and Yaden, 2022; Bahji et al., 2023; Haikazian et al., 2023).

A system is at criticality when its correlation length (the distance within which the value at one point affects the value at another point) diverges (Binney et al., 1992; Cardy, 1996). This leads to a vast repertoire of spatiotemporal fluctuations over many scales (scale invariance), rather than a statistically simpler repertoire where fluctuations tend to take a particular size due to a finite correlation length. The human brain has been suggested to operate in the proximity of criticality (Bak et al., 1987; Cocchi et al., 2017). Neuronal oscillations in the proximity of criticality exhibit complex temporal patterns characterized by slowly decaying temporal auto-correlations of power-law form. These long-range temporal correlations (LRTC) represent memory of past activity in the signal and provide functional advantages for neuronal information processing (Bak, 1996; Shew and Plenz, 2013; Avramiea et al., 2020, 2022). The preservation of LRTCs is associated with normal brain function (Beggs, 2008) and brain maturation is associated with increases in LRTC from childhood to early adulthood (Smit et al., 2011). Alterations in LRTC have also been found to be modulated by genetics (Linkenkaer-Hansen et al., 2007), sleep (Kantelhardt et al., 2015), sleep deprivation (Meisel et al., 2017), anesthesia (Krzemiński et al., 2017), meditation (Irrmischer et al., 2018), and attention (Walter and Hinterberger, 2022). LRTCs imply that past brain activity continues to influence future activity for a long time. Rather than dropping off quickly (e.g., exponentially), the effect of past activity slowly diminishes, providing a form of memory in the system that lasts over large timescales, potentially contributing to the computational abilities of normal waking consciousness (Beggs, 2008; Bruining et al., 2020) and reminiscent of the temporal unfolding of self-referential processing, which maintains coherence over long timescales.

Detrended fluctuation analysis (DFA) assesses the presence of LRTCs in time-series data, and hence the degree to which a signal is consistent with the underlying system being near or far from criticality (Peng et al., 1995; Linkenkaer-Hansen et al., 2001; Hardstone et al., 2012; Poil et al., 2012). While DFA provides information on distance from criticality, the functional excitatory/inhibitory ratio (fE/I ratio; Bruining et al., 2020) allows a distinction between inhibition-dominated (subcritical) and excitation-dominated (supercritical) regimes, thus distinguishing the directionality of criticality signatures observed with DFA (Bruining et al., 2020).

Here, we quantify markers of criticality in two EEG-DMT datasets using DFA and the fE/I ratio, and compare the resulting changes with dissolution of the sense of self, a hallmark of high-dose psychedelic experience with potential therapeutic relevance (Griffiths et al., 2006; Nour et al., 2016; Roseman et al., 2018; Kałużna et al., 2022). Self- or ego-dissolution indicates a breakdown of the coherent sense of self typical of everyday conscious experience (without necessarily implying an absence of phenomenal content). Here, the subjective experience of “self-dissolution” is measured through the relevant questionnaire item “I experienced a disintegration of my usual sense of self or ego.” We find that (1) DMT produces a shift *away* from criticality in alpha and adjacent frequency bands, (2) the observed shift correlates with the ratings for the subjective experience of self-dissolution, and finally (3) the observed shift is in the direction of subcritical regimes. Together, our findings provide novel insights into the mechanism of psychedelic action in the human brain and the neural correlates of the self in normal waking and altered states of consciousness.

## Materials and Methods

### Participants and experimental procedures

For this study the EEG data from two placebo-controlled, single-blind, within-participant studies (Timmermann et al., 2019, 2023b) were combined resulting in 27 healthy participants (12 female, mean age = 34.1, SD = 8.7) after removing participants with unusable EEG data due to muscle and movement artifacts. An initial screening visit to the Imperial College Clinical Research Facility (CRF) assessed physical and mental health to ensure suitability of participants. Exclusion criteria included: being under 18 years old, MR contraindications, no previous psychedelic experiences, an adverse reaction to a psychedelic, a history of psychiatric or physical illness rendering participants unsuitable for participation, a family history of psychotic disorder, or excessive use of alcohol or drugs of abuse. All participants provided written informed consent for participation in the study. Both studies were approved by the National Research Ethics Committee London—Brent and the Health Research Authority and was conducted under the guidelines of the revised Declaration of Helsinki (2000), the International Committee on Harmonization Good Clinical Practices guidelines, and the National Health Service Research Governance Framework. Imperial College London sponsored the research conducted under a Home Office license for research with Schedule 1 drugs.

Study 1 (12 participants from the final sample) was a dose-finding study where participants always received placebo (saline) on a first visit and DMT on a second visit one week after, while EEG recordings took place. The doses administered were as follows: two participants received 7 mg, three participants received 14 mg, one participant received 18 mg, and 4 participants received 20 mg of DMT fumarate—for full recruitment procedure and protocol (Timmermann et al., 2019). In study 2 (15 participants from the final sample), participants received 20 mg of DMT fumarate and placebo (saline) in a counterbalanced order in visits separated 2 weeks apart—for full recruitment procedure and protocol (Timmermann et al., 2023b). For both studies, EEG recordings occurred at baseline (prior to administration) and for 20 min after intravenous administration of DMT fumarate, which was performed during 30 s and followed by a 15-s flush of saline. If participants partook in both studies, we only used the recordings corresponding to their participation in the second study to increase homogeneity in the doses. Participants attended the experimental sessions at the National Institute of Health Research Imperial CRF for Study 1, and the clinical imaging facility (CIF) at Imperial College London for Study 2.

For both studies, following EEG recordings and once subjective effects subsided, participants completed questionnaires and visual analog scales designed to assess the subjective effects experienced during the administration of DMT (see Timmermann et al., 2019, 2023b for full procedures). Of these metrics, we selected 5 visual analog scales for correlations with our EEG results, as these reflect central features of subjective experience we hypothesized are susceptible to changes in LRTC and changes in criticality: (1) the sense of self (“I experienced a disintegration of my usual sense of self or “ego”), (2) the sense of time (“my sense of time was altered”), (3) the sense of space (“my sense of size or space was altered”), (4) cognition (“my thoughts wandered freely”). Additionally, we assessed how LRTC related to generic subjective effects induced by DMT (“how intense was the drug experience”).

### EEG acquisition and preprocessing

The same EEG setup was used for both studies. Data were collected from 31 scalp locations in accordance with the 10–20 system using an MR-compatible BrainAmp MR amplifier (BrainProducts) and a compatible cap (BrainCap MR; BrainProducts GmbH). The system used FCz as the reference for all electrodes, and AFz was the ground electrode. Additionally, an ECG channel was used to capture heart rate recordings. For both recordings data were visually inspected, and segments of data containing muscle artifacts, head motion, and other gross artifact were removed prior to cleaning using independent component analysis (see Timmermann et al., 2019, 2023b for a full list of preprocessing details).

### Quantifying LRTC

DFA measures the scale invariance in temporal correlations, which is reflected by power-laws in fluctuation sizes, a marker of criticality. DFA proceeds from an analogy between the signal (e.g., alpha band amplitude envelopes) and fractional Gaussian noise; validated by the linearity of the log-log fit in fluctuation scalings over a range of timescales. A DFA exponent (slope of the log-log linear fit) of 0.5 (analogous to white noise) indicates no LRTC, indicative the underlying system is far from criticality; a DFA exponent near 1 (analogous to pink noise) is consistent with the system being near criticality, and between 0.5 and 1 indicates a degree of LRTCs increasingly proximal to a potential critical state.

The relationship between entropy, complexity, and criticality is not trivial. In the present context, entropy refers to the unpredictability or randomness of a signal. A white-noise signal exhibits maximal entropy, due to its unpredictability and lack of temporal correlations (no LRTCs; DFA exponent near 0.5), thus reflecting minimal statistical complexity, as it lacks any temporal structure. Colored noise has lower entropy, as its structured temporal correlations make it more predictable (LRTCs, DFA exponent > 0.5), and is statistically more complex, due to the rich variety of patterns elicited by temporal correlations over a wide range of timescales. Hence, in the context of DFA, entropy and complexity are by definition inversely related (Rosso et al., 2007; Zanin and Olivares, 2021). Higher DFA exponents indicate signals with temporal structure over long timescales, higher complexity, lower entropy, potentially proximal to criticality; lower DFA exponents indicate signals with little or no temporal structure, lower complexity, higher entropy, farther away from criticality. In statistical descriptions, as is the case here, entropy can increase away from criticality in *both* directions, as dynamics become increasingly unstructured and white-noise-like. As complexity is generally maximal at criticality due to the range of potential spatiotemporal patterns, these distinctions imply that entropy-complexity-criticality relationships depend on the theoretical framework, and on assumptions on the underlying system (Rosso et al., 2007; Zanin and Olivares, 2021).

**Figure 1.**
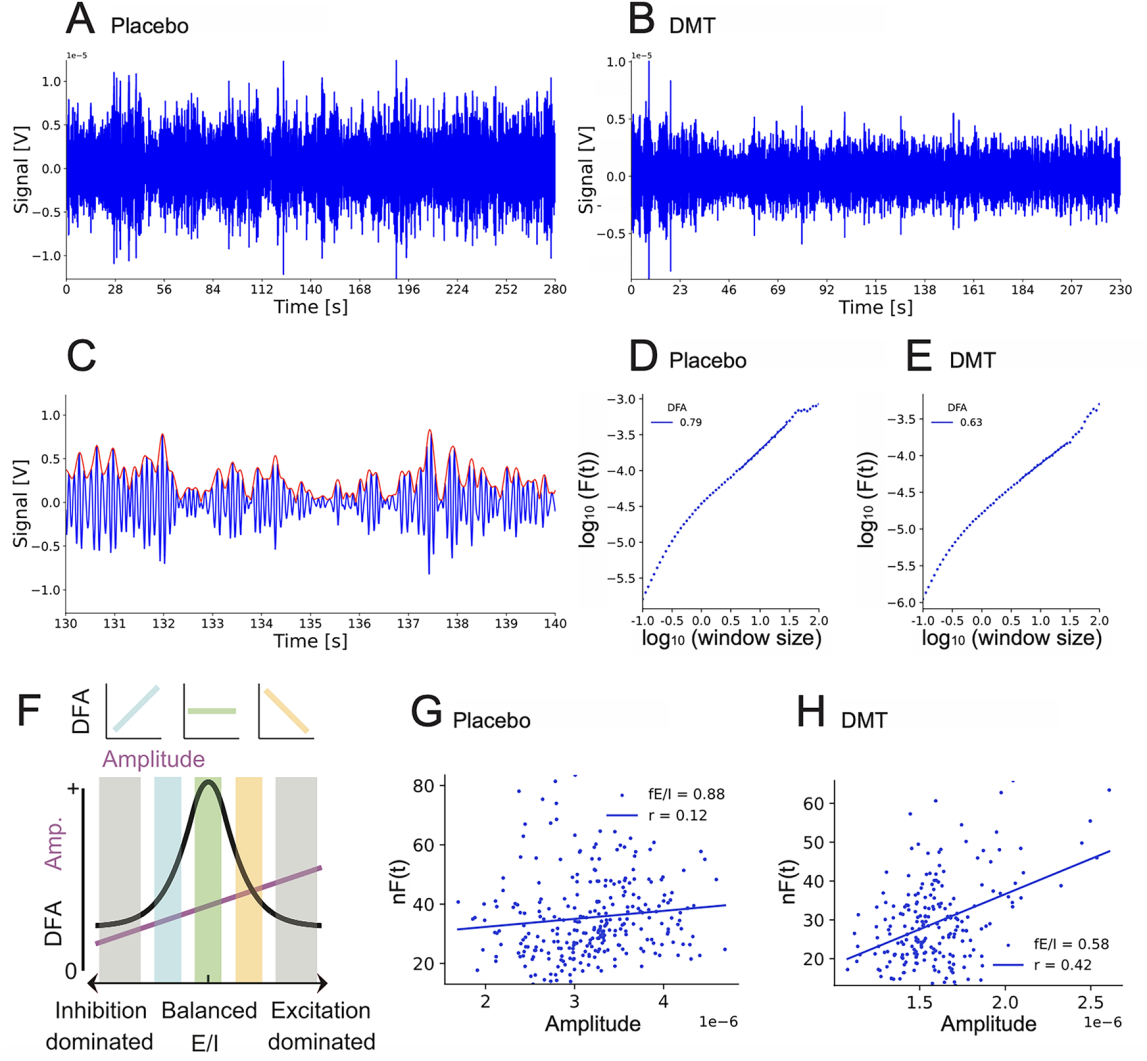
Assessing the criticality of neuronal oscillations under DMT and placebo. Examples shown of EEG from Pz filtered in the alpha-frequency range (8–13 Hz) during the placebo condition (***A***) and during DMT condition (***B***). Note the change to more homogeneous variation in amplitudes during DMT compared to placebo (especially after approx. 20 s, which corresponds to the onset of effects). ***C***, Amplitude of 10 s alpha oscillations is shown in blue and the amplitude envelope in red. In detrended fluctuation analysis (DFA), amplitude modulations from placebo (***D***) and DMT (***E***) are plotted against window size in double-logarithmic coordinates to obtain DFA exponent, a marker for proximity to criticality. ***F***, The DFA exponent cannot distinguish sub- from super-critical activity, unless combined with the functional excitatory/inhibitory ratio (fE/I). fE/I of an example placebo signal (***G***) exhibits expected slight subcriticality close to the critical point, while the fE/I of DMT signal (***H***) shows a shift toward more subcriticality.

Here we assessed LRTC and E/I with the Neurophysiological Biomarker Toolbox written in Python, github links available at (Diachenko et al., 2024). Several measures of E/I balance have been proposed (Ahmad et al., 2022), including LRTC, which is a robust empirical feature of oscillations associated with E/I balance in computational models (Linkenkaer-Hansen et al., 2001, 2007; Monto et al., 2007; Poil et al., 2012). DFA is used to assess LRTC in the signal in the time scales of interest via a DFA exponent. A DFA exponent of 0.5 indicates an uncorrelated random signal (i.e., absence of LRTC), whereas the value >0.5 indicates the presence of positive auto-correlations and their strength. To quantify the strength of LRTC in the amplitude modulation of the EEG oscillations, we first extracted the amplitude envelope using band-pass filters [finite-impulse-response (FIR)-filter], Blackman window with transition bandwidth of 1 Hz (Fig. 1*A,B*) and the Hilbert transform (Fig. 1*C*). Next, the root-mean-square fluctuation of the integrated and linearly detrended signals, *F(t)*, was calculated as a function of time window size, *t* (with an overlap of 50% between windows) and plotted in double-logarithmic coordinates (Fig. 1*D,E*). The DFA exponent is the slope of the fluctuation function *F(t)* in a given interval, which was set to 2 to 30 s for the alpha band.

### Quantifying fE/I

The fE/I algorithm was developed based on an extended version of the critical oscillations computational model of neuronal oscillations (Poil et al., 2012) which mimics the signals observed in human M/EEG recordings. In fE/I, the E/I ratio is estimated from the windowed co-variation of the average amplitude and amplitude modulation (the temporal auto-correlation structure) of frequency-specific activity, in the presence of significant LRTC in the signal (Fig. 1*G,H*), that have been associated with E/I balance in computational models (Poil et al., 2012). It estimates the temporal structure of oscillation amplitude and reflects the level of criticality in the network. DFA is used to assess LRTC in the signal in the time scales of interest via a DFA exponent (Fig. 1). The DFA value of 0.5 indicates an uncorrelated random signal (i.e., absence of LRTC), whereas the value >0.5 indicates the presence of positive auto-correlations and their strength. Taken alone, however, the DFA exponent cannot distinguish sub- from super-critical activity (Fig. 1*F*). Neither can the amplitude of oscillations, which changes monotonously with E/I balance, tell where the critical point is. The combination of the two, on the other hand, can be used to tell apart sub-, critical, and super-critical dynamics (Fig. 1*F*). The correlation of amplitude and DFA is positive for a network operation in a slightly subcritical state (Fig. 1*F* left), zero in a critical state (Fig. 1*F*, middle), and negative in a slightly supercritical state (Fig. 1*F*, right). Given that networks operating in these regimes exhibit co-variation in amplitude and temporal structure, we can use a sliding-window approach to quantify this co-variation and, thus, infer the E/I balance of the underlying networks. Of note, when networks are far from criticality as reflected in DFA exponents <0.6, there is no correlation between the co-variation of amplitude and temporal structure and, therefore, the DFA exponent of 0.6 is used as a threshold to compute the fE/I ratio (Bruining et al., 2020).

Given the presence of LRTC in the signal reflected by the DFA exponent >0.6, fE/I is then computed by correlating the amplitude and LRTC in short windows. Windowed LRTC, in this case, is estimated through the normalized fluctuation function which serves as a reliable proxy of the DFA exponent on short time scales. Overall, the main steps of the fE/I algorithm are as follows (Bruining et al., 2020):

1. The signal is bandpass-filtered in the desired frequency range using a FIR filter.
2. The amplitude envelope of the filtered signal is extracted using Hilbert Transform.
3. The signal profile is calculated and segmented into 80%-overlapping 5-s windows.
4. The windows are normalized using the mean of the amplitude envelope calculated per window.
5. Subsequently, the normalized windows are detrended.
6. The normalized fluctuation function for each window is computed as the root-mean square fluctuation of the detrended amplitude-normalized signal profile.
7. Finally, the fE/I value is obtained by subtracting the Pearson correlation between the windowed normalized fluctuations and windowed amplitudes from 1. The fE/I is set to NaN (i.e., missing) if DFA exponent does not exceed the DFA threshold of 0.6.

### Statistical analyses

The EEG analysis was performed per channel with a non-directional paired *t*-test (level: *p* < 0.05), with average DFA across statistically significant electrodes reported. Changes in DFA and fE/I were calculated between the placebo and DMT condition to avoid order effects, while the correlations with changes in subjective experience was performed with changes from DMT baseline to DMT after injection, to minimize noise. Due to the continuous nature of subjective experience, the parametric Pearson’s correlation coefficient was used to test for correlations with experience. To prevent chance-level effects, we used the Benjamini Hochberg false discovery rate (FDR) multiple comparison correction method (Benjamini and Hochberg, 1995) as post hoc test with FDR adjusted *q*-values of <0.10 within a specific frequency band.

## Results

### DMT shifts dynamics of theta, alpha, and beta oscillations away from criticality; toward increased entropy and reduced complexity

**Figure 2.**
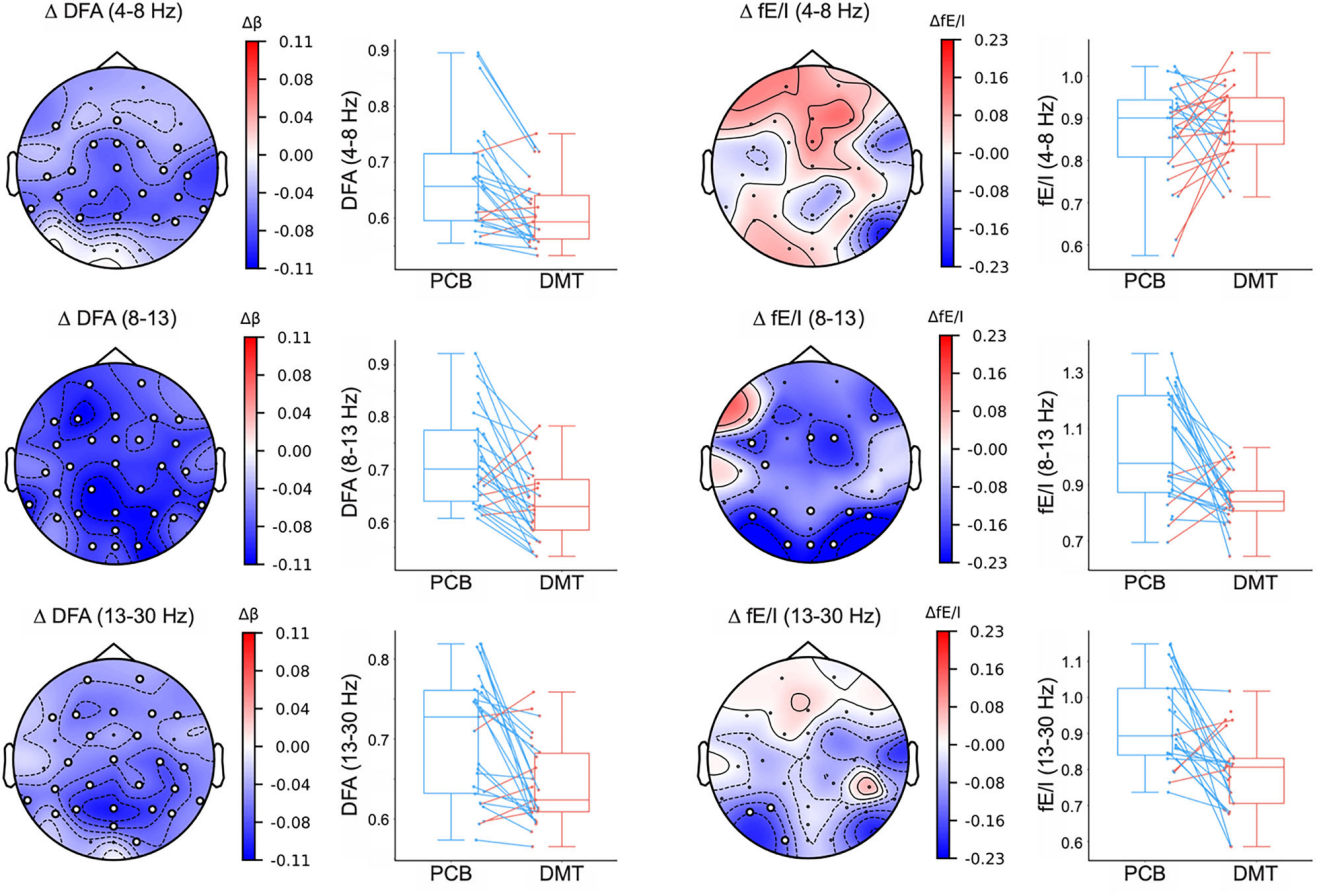
Brain oscillations under DMT in multiple frequency bands shift away from criticality, and toward (more entropic) subcritical regimes. Left, DFA exponents show statistically significant decreases in multiple frequency bands, throughout the scalp (electrodes with circles). This indicates a shift away from critical dynamics in these frequency bands. Right, fE/I estimates show statistically significant decreases in the alpha band for occipital and parietal regions, and in the beta band for left-occipital region (electrodes with circles). This indicates a shift toward more entropic, subcritical dynamics in these frequency bands and regions. Data points in box plots represent the mean of statistically significant electrodes per-participant.

In order to investigate the impact of DMT on the criticality of brain oscillations, we quantify the presence of LRTC following DMT and placebo administration in the same participants. We computed the DFA exponent as an index of LRTC. Paired-samples *t*-tests were conducted to compare the difference in DFA between placebo and DMT conditions for the theta, alpha and beta band. Compared to placebo, DMT-induced a significant decrease of DFA in theta ( $\Delta {\text{DFA}}_{\theta }=-0.06$; *p* = <.0001), alpha ( $\Delta {\text{DFA}}_{\alpha }=-0.09$; *p* = <0.0001) and beta ( $\Delta {\text{DFA}}_{\beta }=-0.06$; *p* = 0.0004). These observed reductions in DFA exponents were widespread throughout the scalp (Fig. 2, left column), and are robust across subjects (Fig. 2, left column boxplots). In sum, we find that DMT shifts the dynamics of brain oscillations in alpha and adjacent frequency bands *away* from criticality.

As implied above, DFA exponents also provide information on the entropy and complexity of brain signals. A DFA exponent of 1.0 represents a pink-noise-like signal (*1/f* spectrum), while a DFA exponent of 0.5 represents a white-noise-like signal (flat spectrum). Exponents with intermediate values represent the spectrum of fractional Gaussian noises between these two extrema. In this context, entropy and complexity are inversely related (Rosso et al., 2007; Zanin and Olivares, 2021). A white-noise-like signal has higher entropy but lower complexity, while a pink-noise-like signal has lower entropy but higher complexity. As such, a reduction in DFA exponent estimates, in the range between 0.5 and 1, such as that we observe here, indicates a shift away from a more complex, less entropic, pink-noise-like signal toward a less complex, more entropic, white-noise-like signal. In sum, our findings imply that brain oscillations in alpha and adjacent frequency bands under DMT become less complex, but more entropic, throughout the brain.

### DMT shifts dynamics of alpha and beta oscillations toward subcritical regimes

DFA does not provide information on the direction of the criticality-shifts it quantifies. Starting from a near-critical point, both shifts toward subcritical and supercritical dynamics are characterized by reductions in DFA exponents, such as those we observe here. To distinguish the directionality of the shift we observed, we computed a metric recently introduced for this purpose, the fE/I ratio (Bruining et al., 2020; Diachenko et al., 2024). Paired-samples *t*-tests were conducted to compare the difference in fE/I between the placebo and DMT condition. Compared to placebo, DMT-induced a statistically significant decrease of fE/I for the alpha band ( $\Delta {\text{\textbackslash{},fE/I}}_{\alpha }=-0.18$; *p* = .0003), which were especially pronounced in parietal and occipital electrodes. In the beta band the reduction only reached significance in the left-occipital region ( $\Delta {\text{\textbackslash{},fE/I}}_{\beta }=-0.14$; *p* = .0005; Fig. 2, right column). In sum, we find evidence that the observed criticality-shifts in alpha and beta bands are in the subcritical direction.

### Criticality-shifts elicited by DMT correlate with the experience of self-dissolution

**Figure 3.**
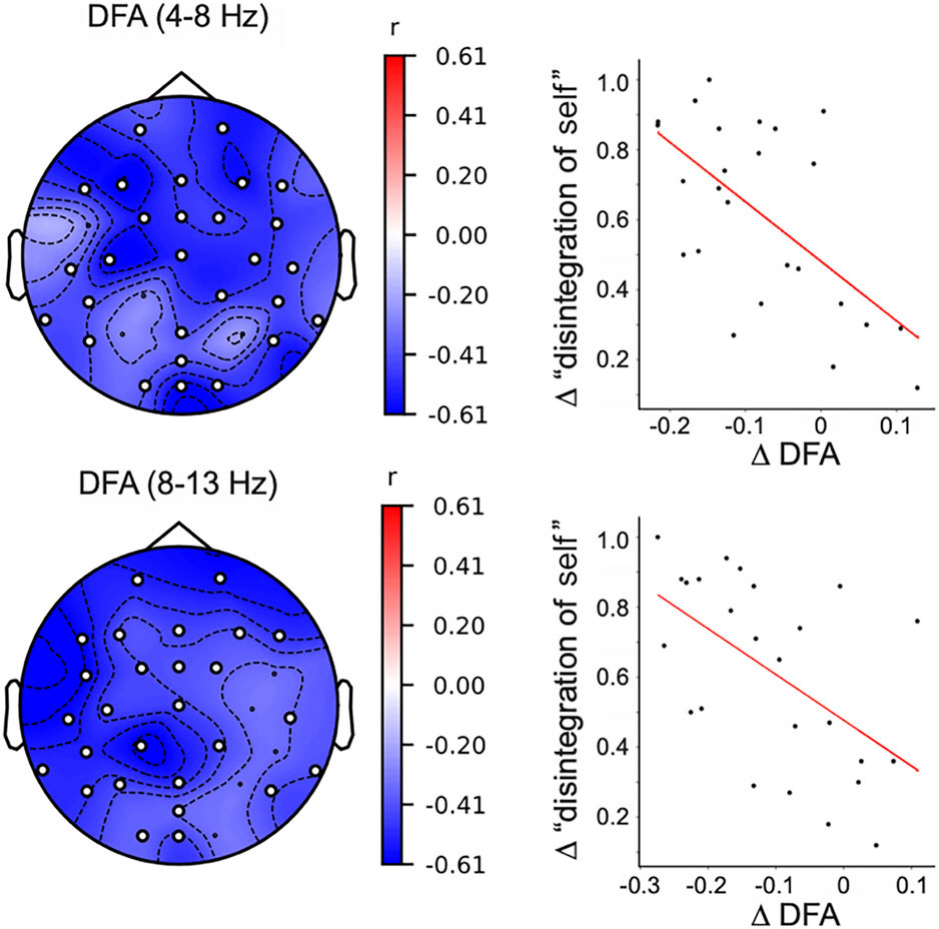
Criticality-shift correlates with self-dissolution. The change in DFA exponent in theta and alpha bands induced by DMT correlates with self-dissolution experience. Left column, the correlation between self-dissolution experience and DFA is statistically significant (filled circles) for electrodes throughout the scalp. Right column, correlation between mean DFA change in statistically significant electrodes of each participant and ratings of self-dissolution.

To test whether shifts in criticality of neuronal oscillations measured with DFA and the fE/I ratio were associated with DMT-induced alterations in self-related processing, we performed correlations with four items of the Visual Analog Scales (VAS) related to 3 elements related to disruptions in self-related processing as well as a generic item broadly assessing disruptions in the sense of self (see Methods). We found statistically significant correlations between criticality shifts (measured with DFA estimates) and the generic item of self-disruption (measured with the item: “I experienced a disintegration of my usual sense of self or ego”) in the theta [*r*(25) = −0.61, *p* = 0.001] and alpha bands [*r*(25) = −0.56, *p* = 0.005]. The correlation were statistically significant for most electrodes across the scalp (Fig. 3). In sum, we find that the magnitude of the shift away from criticality elicited by DMT in theta and alpha bands relates to the disruptions in self-related processing.

## Discussion

Here, we investigated the effects of DMT, a classic psychedelic, on markers of criticality, and their relation to subjective experience. We found statistically significant shifts *away* from criticality for oscillations in theta, alpha, and beta bands, implying a reduction in complexity and an increase in entropy. We found statistically significant shifts toward subcritical regimes, in occipital and parietal alpha, and left-occipital beta bands. Finally, we found statistically significant correlations between DFA reductions in alpha and theta bands and ratings of self-dissolution. Our findings provide novel information on the effects of DMT (shifts *away* from criticality, and toward a more entropic, less complex subcritical regime), in alpha and adjacent frequency bands. Furthermore, our findings demonstrate a relationship between criticality-shifts and the disruption of the self, providing novel candidates of neural correlates of self-related processing in the human brain.

Classic psychedelics act through the (excitatory) 5-HT2A receptor (Vollenweider and Preller, 2020). Previous studies have reported increases in measures of entropy under psychedelics (Schartner et al., 2017; Timmermann et al., 2019, 2023b; Pallavicini et al., 2021). Shifts away from criticality have also been observed in states of anesthetic unconsciousness and meditation, which certainly have profound differences with psychedelic states (Irrmischer et al., 2018; Bruining et al., 2020). On one hand, psychedelics generally elicit states of high phenomenal richness; on the other, anesthetics generally elicit states of little or no phenomenal experience. If the effects of anesthetics and psychedelics are taken to be completely distinct, our observation of weaker LRTCs and shifts toward subcritical dynamics in alpha and adjacent frequency bands may appear puzzling. However, high-dose psychedelic and anesthetic states may also share notable features. In particular, the loss or severe disruption of the coherent, self-referential stream of thought, unfolding over medium-long timescales, which is a cornerstone of daily waking experience. Our findings suggest that reduction in DFA and fE/I metrics in alpha and adjacent frequency under DMT might specifically track the disruption of this process shared by high-dose psychedelic states as well as anesthetic or deep meditation states. Indeed, we believe that the measures employed in our study selectively capture the shared nature of the latter similarity between otherwise distinct states of consciousness. As such, our study represents a step forward toward understanding the neural correlates of multidimensional consciousness.

Alpha frequencies have been implied as top-down carriers of predictive models instantiated by high-level regions and networks (Alamia and VanRullen, 2019). Theoretical frameworks and empirical evidence have indeed implicated reductions of alpha oscillations and default-mode network (DMN) connectivity (a system known to relate to alpha oscillations), as key players in psychedelic effects (Carhart-Harris and Friston, 2019). In fact, both systems have been found to be significantly dysregulated under DMT in an interrelated fashion (Timmermann et al., 2023b). However, alpha oscillations may also relate to activity in low-level sensory regions, which coexist in a dialogical interplay with high-level ones (Aqil and Roseman, 2023). Alpha oscillations and DMN activity are strongly suppressed by visual content, which is intensely present during DMT states (Timmermann et al., 2019). In fact, DMT-induced dysregulation of alpha oscillations has been found to significantly correlate with the intensity of visual experience (Timmermann et al., 2019). Further work is needed to clarify the role of alpha oscillations in the interplay between low and high-level cognition.

We speculate that supercritical regimes under DMT and other classic psychedelics (and the corresponding richness of phenomenal content) may be apparent in high frequency oscillations (high gamma), which are not accessible here because of experimental limitations. Changes of this nature have been observed in animal models of psychedelics (Yu et al., 2023). Alternatively, it is possible that theta oscillations may reflect supercritical regimes under DMT, as increases in theta have been found to be correlated with the visual experience induced by DMT (Timmermann et al., 2019). Future studies should narrow the EEG analysis to moments of experience in which increases in content richness are present to advance these questions. This could be achieved by employing methodological paradigms that carefully match subjective experience with specific moments of the psychedelic state (Timmermann et al., 2023a).

Aside from mathematically-defined theoretical systems, it is generally not possible to strictly confirm that a real-world system is at criticality. Rather, it is possible to test for signatures that are consistent with critical dynamics. One such signature is the presence of LRTCs quantified by the DFA exponent. The DFA exponent is a direct generalization of the Hurst exponent. However, DFA is considered more robust to non-stationarities and trends in the data and a wealth of results implicates its ability to measure functionally relevant brain properties (Linkenkaer-Hansen et al., 2001; Poil et al., 2012). Thus, we chose DFA over other alternative measures such as the Hurst exponent. A reduction in alpha oscillatory power does not automatically imply a shift away from criticality (Linkenkaer-Hansen et al., 2007). The reduction in DFA and fE/I measures we observe therefore represent a novel finding, distinct from the previously known reduction in power, and providing evidence of a shift toward subcritical regimes in alpha and neighboring frequency bands elicited by DMT.

Self-referential processing is thought to encompass functional connections between widespread brain regions. Long-range amplitude coupling is optimized for brain networks near criticality (Avramiea et al., 2022). Shifts in DFA have previously been found in deep meditation and anesthetic states, which also present a reduced or absent self-referential processing (Krzemiński et al., 2017; Irrmischer et al., 2018). Hence, we hypothesized that LRTCs might underlie the coherent, temporally extended stream of consciousness in our daily experience, disrupted in states such as high-dose psychedelics, anesthesia, or deep meditation (despite these states differing on other dimensions, such as richness of phenomenal content). As such, we carried out correlations only with selected questionnaire items that related to disruptions of the sense of self. A limitation of our study is that we did not ask participants to explain what they understood a particular questionnaire item to mean. This could be a useful consideration for future studies, in order to reduce potential ambiguity.

The entropic brain hypothesis (Carhart-Harris et al., 2014; Carhart-Harris, 2018; Carhart-Harris and Friston, 2019) suggests that during psychedelic states (1) brain entropy increases and (2) brain dynamics move closer to criticality, starting from slightly subcritical states during normal waking consciousness. Here, we find that (1) brain entropy increases during psychedelic states, and (2) alpha and beta oscillations move in the subcritical direction. Hence, our results are consistent with the entropic brain hypothesis insofar as entropy increases; but not consistent insofar as we observe shifts *away* from criticality. The discrepancy can be explained by the different relationships between entropy and criticality in different systems. In the context of the original entropic brain hypothesis, entropy is assumed to increase monotonically from subcritical, to critical, to supercritical states, as indeed can be the case in some dynamical systems. However, in this context, a decrease in DFA exponent indicates a shift from a more complex, less entropic pink-noise-like signal to a more entropic, less complex white-noise-like signal (Rosso et al., 2007; Zanin and Olivares, 2021). We suggest that subcritical shifts in alpha and neighboring frequencies might relate to the disruption of self-referential processing, as indeed they correlate with the intensity of self-dissolution. We speculate that high gamma oscillations (>40 Hz) might instead display shifts toward criticality or beyond, into supercritical regimes, potentially indexing the richness and complexity of phenomenal content during psychedelic states. This is consistent with recent findings showing how increases in complexity in psychedelic states are mostly attributed to high frequency components of brain signals (Mediano et al., 2023). Our findings suggest a nuanced, potentially frequency-dependent relationship between criticality of brain oscillations and different dimensions of psychedelic subjective experience, moving beyond global measures of brain entropy and a generic notion of “psychedelic state.”

In sum, we find that DMT alters the criticality signatures of brain oscillations, and that the observed shifts in criticality correlate with the subjective experience of self-dissolution. In particular, we find that DMT shifts oscillations in alpha and adjacent frequency bands away from criticality and toward subcritical regimes characterized by increased entropy but reduced complexity. Weak, subcritical LRTCs in alpha and adjacent bands may be a potential shared neural correlate of the disruption of conscious self-related processing, common both to high-dose psychedelic experiences, deep meditation states, and anesthesia. Overall, our findings provide novel information on the effects of psychedelics on criticality, entropy, and complexity of brain oscillations, and their relation with subjective experience.
